# Structural and Functional Characterization of a New Bacterial Dipeptidyl Peptidase III Involved in Fruiting Body Formation in Myxobacteria

**DOI:** 10.3390/ijms24010631

**Published:** 2022-12-30

**Authors:** Si-Bo Chen, Han Zhang, Si Chen, Xian-Feng Ye, Zhou-Kun Li, Wei-Dong Liu, Zhong-Li Cui, Yan Huang

**Affiliations:** 1 Key Laboratory of Agricultural Environmental Microbiology, Ministry of Agriculture and Rural Affairs, College of Life Sciences, Nanjing Agricultural University, Nanjing 210095, China; 2 Industrial Enzymes National Engineering Laboratory, Tianjin Institute of Industrial Biotechnology, Chinese Academy of Sciences, Tianjin 300308, China

**Keywords:** *Corallococcus* sp. EGB, dipeptidyl peptidase III, crystal structure, fruiting body development

## Abstract

Dipeptidyl peptidase III (DPP III) is a zinc-dependent enzyme that specifically hydrolyzes dipeptides from the N-terminal of different-length peptides, and it is involved in a number of physiological processes. Here, DPP III with an atypical pentapeptide zinc binding motif (HELMH) was identified from *Corallococcus* sp. EGB. It was shown that the activity of recombined *Co*DPP III was optimal at 50 °C and pH 7.0 with high thermostability up to 60 °C. Unique to *Co*DPP III, the crystal structure of the ligand-free enzyme was determined as a dimeric and closed form. The relatively small inter-domain cleft creates a narrower entrance to the substrate binding site and the unfavorable binding of the bulky naphthalene ring. The ectopic expression of *Co*DPP III in *M. xanthus* DK1622 resulted in a 12 h head start in fruiting body development compared with the wild type. Additionally, the A-signal prepared from the starving DK1622-*Co*DPP III rescued the developmental defect of the *asgA* mutant, and the fruiting bodies were more numerous and closely packed. Our data suggested that *Co*DPP III played a role in the fruiting body development of myxobacteria through the accumulation of peptides and amino acids to act as the A-signal.

## 1. Introduction

Dipeptidyl peptidase III (DPP III, EC 3.4.14.4) is a zinc-metallopeptidase of the M49 family, which sequentially cleaves dipeptides from the N-termini of the oligopeptide ranging from three to ten amino acid residues [1]. DPP III is assumed to play a role in the final stages of cytosolic peptide turnover. Thus, DPP III affects various physiological processes in mammals, such as pain and blood pressure modulation, endogenous defense against oxidative stress, and cataractogenesis [2,3,4,5]. It also was suggested that DPP III is an activator in the Keap1-Nrf2 signaling pathway and is involved in cancers due to its overexpression in ovarian malignant tissues, endometrial carcinoma cells, and glioblastoma cells [6,7]. DPP III has been identified as a potential drug target, and efforts toward the design and synthesis of inhibitors are underway [8,9,10]. 

Although DPP III was previously thought to be merely distributed in eukaryotic cells, DPP III has now also been found in various different organisms thanks to the availability of newly sequenced genomes [11,12,13,14,15,16,17,18,19]. Although they have low homology within all the phylogenetic domains, all their amino acid sequences harbor two conserved sequence regions of the M49 family, including the signature hexapeptide zinc-binding motif HEXXGH and EEXR(K)AE(D) [20]. However, new members of DPP III have been discovered which possess a reduced pentapeptide instead of a hexapeptide. Moreover, they can be divided into two groups; one is distributed in plants and contains a pentapeptide HECCH and an extra Nudix box, named Nudix hydrolase 3 with dual activity. Recently, samples of Nudix hydrolase 3 from *Physcomitrella patens* (PpND) and *Arabidopsis thaliana* (AtND) have been cloned and characterized, and it was shown that the insertion of the active-site motif to mimic hexapeptide could result in 57-fold decreased activity towards the diarginyl-2-naphthylamide substrate [16]. The other new member is bacterial DPP III with a shorter sequence length compared to its eukaryotic counterparts, which have a pentapeptide HEXXH. Only DPP III from thermophilic bacteria *Caldithrix abyssi* (*Ca*DPP III) was investigated in terms of its structure and dynamics to provide an explanation for the thermal stability and reduced activity [21].

In addition, the crystal structures of DPP III from humans (*h*DPP III), as well as from *Saccharomyces cerevisiae* (*y*DPP III) and *Bacteroides thetaiotaomicron* (*Bt*DPP III), have been determined [22,23,24]. All DPP III display a similar overall fold, consisting of two domains that are separated by a wide-open substrate-binding cleft. Notably, in the presence of ligands, the two domains rotate, and DPP III adopts a closed conformation [25]. Several complex structures of *h*DPP III have shown that the overall binding mode of peptides is conserved, extensive polar contacts of the N-terminal peptide residues and the formation of β-type interactions with the core of the enzyme [26,27]. Based on the different interaction of the carbonyl group of the scissile peptide bond with the zinc ion, it was proposed that efficiently cleaved peptides follow the favored water-mediated hydrolysis mechanism, whereas inhibitory peptides might displace a zinc-bound water molecule, thus following an energetically much less favored anhydride mechanism [28].

In contrast to the extensive information regarding enzymatic properties, crystal structure, and physiological role of eukaryotic DPP III, the prokaryotic orthologs have been much less commonly investigated. To date, only four bacterial DPP III have been biochemically characterized [15,21,29,30]; however, there has been little discussion regarding their physiological function. It is speculated that DPP III might be involved in the generation of free amino acids for the growth of the asaccharolytic bacterium *P. gingivalis* [15], or the generation of health-promoting bioactive peptides for the immunomodulation of the probiotic lactic bacterium *P. acidilactici* [30].

Myxobacteria are one of the bacterial groups that live in soil as a microbial predator with a complex life cycle [31]. They are well-known for their multicellular behaviors which are often associated with morphological changes. Under starvation conditions, some cells aggregate into a multicellular fruiting body, wherein some cells differentiate into environmentally resistant spores [32]. Currently, five classes of mutants have been identified to be deficient in forming fruiting bodies or spores, recognized as *asgA*, *asgB*, *asgC*, *asgD,* and *asgE*. The fruiting body formation depends on these five intercellular signals; among them, A-signal and C-signal have been extensively characterized. It was suggested that the A-signal is an extracellular factor required early in fruiting body development after 2 h starvation, which is composed of a heat labile and a heat stable fraction. The heat labile fraction is a mixture of proteases, and the heat stable fraction consists of amino acids and peptides [33,34]. Regulated proteolysis in bacteria can be generated by proteases or peptidases. Although several extracellular proteases have been purified from *Myxococcus xanthus* [28,35,36,37,38,39,40], no A-signal protease or peptidase has been identified. *Corallococcus* sp. EGB is a member of myxobacteria with a prominent predation ability against plant pathogenic fungi; thus, it must secrete many hydrolytic enzymes. Thereafter, the culture supernatant of strain EGB was analyzed, and we identified a new type of dipeptidyl peptidase III (*Co*DPP III) from the extracellular proteins. Here, for the first time, we biochemically and functionally described DPP III from *Corallococcu*s sp. EGB.

## 2. Results and Discussion

### 2.1. Cloning and Sequence Analysis of CoDPP III

Extracellular proteins of *Corallococcus* sp. strain EGB were analyzed using matrix-assisted laser desorption/ionization–time-of-flight (MALDI-TOF) peptide mass fingerprinting (PMF). Among the stained gel bands, three peptide fragments of a specific band were detected and shared 100% identity with the peptides of a hypothetical protein, COCOR_06584 (GenBank accession no. AFE07001.1) from *C. coralloides* DSM 2259 (Figure S1). Thereafter, the corresponding gene in *Corallococcus* sp. strain EGB was cloned; it contained a 1719 bp open reading frame encoding 572 amino acids, with a predicted signal peptide (residues 1 to 22). The molecular mass and *pI* were calculated to be 62.48 kDa and 7.9, respectively. The calculated molecular mass of *Co*DPP III was much higher than the molecular mass of the native protein. This was also the case for the identified AmyM from the supernatant, the molecular mass of calculation and native protein of which was 55.5 kDa and 43 kDa, respectively [41]. In view of the reports regarding various proteases from myxobacteria, the smaller molecular mass of the native protein might have been caused by proteolytic cleavage.

**Figure 1:**
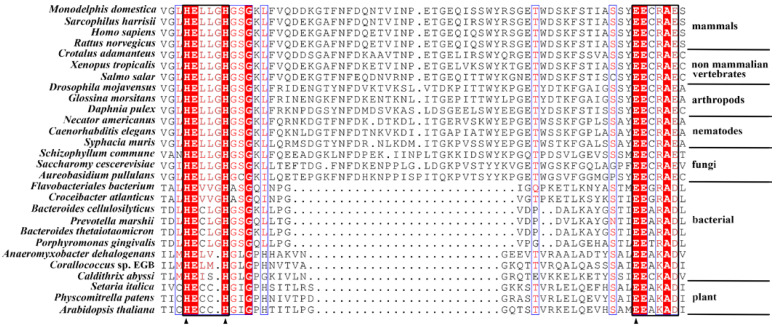
A section of a multiple sequence alignment of DPP III from various species. The organisms and UniProt sequence identifiers are *Monodelphis domestica* (F6ZXH8), *Sarcophilus harrisii* (A0A7N4P2W4), *Homo sapiens* (Q9NY33), *Rattus norvegicus* (O55096), *Crotalus adamanteus* (J3RZ30), *Xenopus laevis* (Q6DE90), *Salmo salar* (B5X435), *Drosophila mojavensis* (B4K999), *Glossina morsitans* (D3TMQ7), *Daphnia pulex* (E9GTX0), *Necator americanus* (W2SLT7), *Caenorhabditis elegans* (G5ECW7), *Syphacia muris* (A0A0N5AXJ5), *Schizophyllum commune* (D8PVF6), *Saccharomyces cerevisiae* (Q08225), *Aureobasidium pullulans* (A0A074XX23), *Flavobacteriales bacterium* (A4AQA4), *Croceibacter atlanticus* (A3U8C5), *Bacteroides cellulosilyticus* (E2NMV7), *Prevotella marshii* (E0NQE0), *Bacteroides thetaiotaomicron* (A0A139KC05), *Porphyromonas gingivalis* (Q7MX92), *Anaeromyxobacter dehalogenans* (Q2IN78), *Corallococcus* sp. EGB (A0A5H1ZR28), *Caldithrix abyssi* (H1XW48), *Setaria italica* (K3YPW2), *Physcomitrella patens* (A9TLP4), and *Arabidopsis thaliana* (Q8L831). The conserved motifs are marked by rectangular boxes. The triangles show the amino acids involved in zinc ion coordination. Dots indicate gaps. Identical amino acid residues in the same position are shaded.

The BLASTP analysis showed that the deduced amino acid sequence shared high identity (>50%) with that of the hypothetical protein, the DNA mismatch repair protein MutT, and the peptidase family M49 in the genome of several myxobacteria. Among the characterized enzymes, the amino acid sequences shared a higher identity with the members of the peptidase family M49, such as Nudix hydrolase 3 from *Arabidopsis thaliana* (32%), DPP III from *Caldithrix abyssi* (*Ca*DPP III, 42.5%), *Bacteroides thetaiotaomicron* (*Bt*DPP III, 14.3%), human (*h*DPP III, 13.7%), and *Saccharomyces cerevisiae* (*y*DPP III, 12.7%). Although it shares a higher identity to Nudix hydrolase 3, it was shown that *Co*DPP III lacks the N-terminal Nudix box (GX_5_EX_7_REUXEEXGU). Multiple alignments of the primary sequences revealed that the deduced amino acid sequence retains the conserved regions in the M49 family, but the typical hexapeptide HEXXGH signature motif is reduced to pentapeptide HEXXH; thus, the cloned gene was named *Codpp III.* The catalytic motif and the second most highly conserved motifs of *Co*DPP III were HELMH and EEAKAD, similar to HECCH and EEAKAD in plants, but different to HEXXGH and EEXRAE/D in mammals, non-mammalian vertebrates, arthropods, nematode fungi, and other bacteria (Figure 1 and Figure S2).

### 2.2. Biochemical Characterization of DPP III

**Figure 2:**
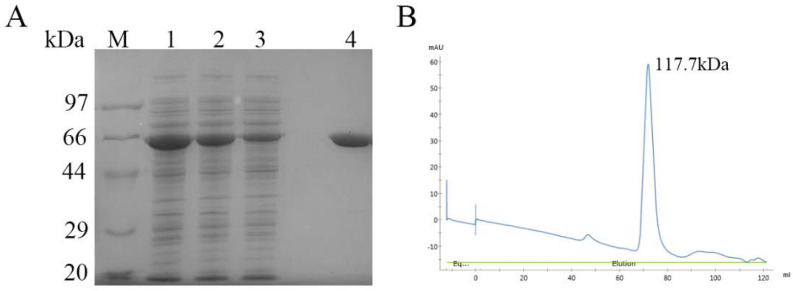
SDS−PAGE (**A**) and size−exclusion gel chromatography (**B**) of purified recombinant *Co*DPP III. M: molecular weight markers; lane 1: the supernatant fraction; lane 2: the precipitant fraction; lane 3: the flowthrough fraction; lane 4: the elution fraction.

Full-length *Co*DPP III was cloned and expressed in *E. coli* BL21(DE3) at high expression levels of 7 mg/mL. Despite its modification with a hexa-histidine affinity tag at the C-terminus, *Co*DPP III could not bind to Ni^2+^-NTA resin. It was supposed that the his-tag might not have been completely exposed on the surface in the process of protein folding. Thereafter, cation exchange chromatography was employed based on the *pI* of *Co*DPP III. Recombinant *Co*DPP III was purified to apparent homogeneity according to SDS-PAGE, with a molecular mass of 62 kDa, and it was eluted as a single peak calculated at 117.7 kDa via size-exclusion gel chromatography (Figure 2 and Figure S3), suggesting its dimeric formation under non-denaturing conditions. The most commonly characterized bacterial DPP III are monomeric enzymes with molecular weight in the range of 72–260 kDa, except that protein purified from *P. acidilactici* is a heterotetramer with different molecular masses: 33.02, 30.31, 25.01, and 19.9 kDa [30]. The presence of a monomer and dimer in preparations of purified yeast DPP III was also reported [42]. A homodimer of 66 kDa and a heterodimer of 60 kDa were also reported in porcine spleen [43] and in *Vigna radiata* [44], respectively.

**Figure 3:**
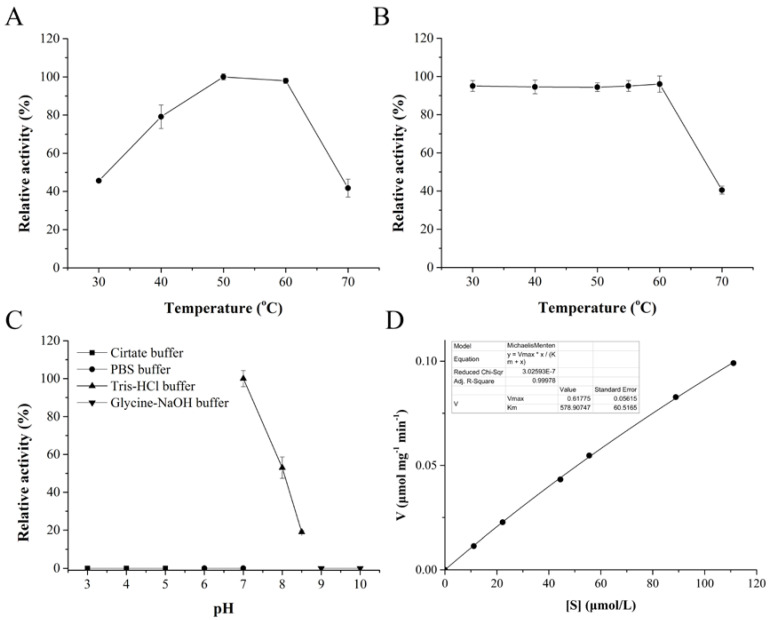
Biochemical properties of *Co*DPP III. (**A**) Optimal temperature, (**B**) thermo stability, (**C**) optimal pH, and (**D**) Michaelis–Menten plot.

The temperature optimum of *Co*DPP III was 50–60 °C, and enzyme activity abruptly declined at 70 °C (Figure 3A). *Co*DPP III was stable up to 60 °C, and 60% of activity was lost at 70 °C (Figure 3B). *Co*DPP III exhibited higher thermal stability than its counterparts, which worked optimally at 37 °C and were almost completely inactivated at 50–55 °C [29,30,45,46], except for *Ca*DPP III from a thermophile, which also displayed its maximum activity at 50 °C [21]. 

Recombined *Co*DPP III was active in the very narrow and slightly alkaline pH range of 7.0–8.5 and exhibited maximal activity at pH 7.0 in Tris-HCl buffer; however, it was inactive in phosphate buffer at the same pH (Figure 3C). The pH profile was consistent with that of *Ca*DPP III, which showed the highest and 20% of activity in pH 7.0 Tris-HCl and PBS buffer, respectively. Moreover, 6% of the activity of *Ca*DPP III was retained in pH 6.0 PBS buffer. It was reported that the ionic strength of the Tris-HCl buffer (optimal I = 0.05) had an influence on the activity of *Bt*DPP III. In addition, the kosmotropic salts significantly decreased in enzyme activity at 0.05 M (sodium citrate > sulfate and acetate). HPO_4_^2-^ as a kosmotropic anion and Na^+^ as a chaotropic cation have been demonstrated to inhibit enzyme activity. The Hofmeister effect might offer a reasonable explanation for the catalytic behavior of DPP III, including the effect of ions on protein–water interactions, the surface pH, the net charge, and the active site modification of the enzyme molecule. Higher optimal pH value of 7.9–8.0 in Tris-HCl buffer were reported for AtND, *Bt*DPP III, and *Pg*DPP III [16,29,30]. *Bt*DPP III retained approximately 20% of activity at pH 7.0 and 9.0. Human and rat DPP III exhibited optimal pH at pH 8.0 and pH 8.8, and at physiological pH (pH 7.4), both enzymes displayed 45% of their maximum activities [47].

**Figure 4:**
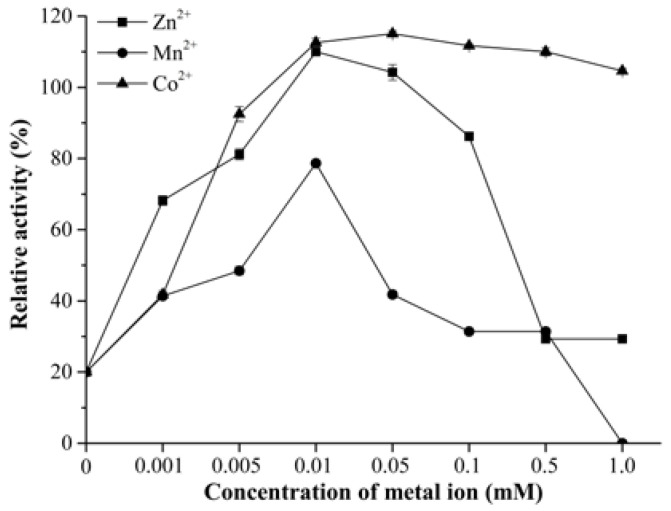
Reactivation of EDTA inhibited activity of *Co*DPP III via metal ions.

As a metalloenzyme, *Co*DPP III was found to be sensitive to metal chelators. The enzyme retained 20% and 1% of activity when using 1 mM and 5 mM EDTA, and it was completely inhibited by 10 mM EDTA. The inhibition of DPP III by EDTA has also been reported by many researchers: rat DPP III, *h*DPP III, *Bt*DPP III, *Pa*DPP III, and *Ca*DPP III retained 23.3%, 14.7%, 3.8%, 39.68%, and 1.4% of activity with 10 mM EDTA, respectively. Inhibition studies demonstrated the difference in the sensitivity of DPP III toward EDTA. It was suggested that the residual activity of ETDA-treated enzymes might have resulted from the existence of a slowly exchanging site on DPP III [47]. The inhibited activity could completely be restored by 0.01 mM Co^2+^ followed by Zn^2+^ and Mn^2+^ (Figure 4), but Mg^2+^, Ba^2+^, and Ca^2+^ had no effect. The enzyme activity was inhibited with a further increase in the concentration of Zn^2+^ and Mn^2+^; however, *Co*DPP III could obtain a high level of activity (77.5%) even at 5 mM Co^2+^. Although Zn^2+^ is indispensable for the activity of native DPP III, the ability to regain enzyme activity through Co^2+^ and Mn^2+^ suggested that certain metal ions might substitute its catalytical role at the active site, partially explained by their similar sizes. 

Under the standard assay condition (Table S1), the activity of *Co*DPP III was almost abolished by the addition of 0.1 mM Cu^2+^, Ni^2+^, and 0.5 mM Zn^2+^. This enzyme is activated by Co^2+^, Mn^2+^, Mg^2+^, Ba^2+^, and Ca^2+^, of which Co^2+^ is the most effective activator. Excess of Mn^2+^ inhibited the metal-depleted *Co*DPP III, but not the zinc-binding enzyme. We suppose that the presence of Zn^2+^ showed a significant protective effect against Mn^2+^ inactivation by maintaining the configuration of active site. In contrast, significant inhibition via 0.2 mM Mn^2+^, Mg^2+^, and Ca^2+^ and activation via Co^2+^ and Cu^2+^ were observed in *Pa*DPP III [30]. Co^2+^ is the activator of all hitherto characterized DPP III. AtND, *Bt*DPP III, and *Ca*DPP III were activated by Co^2+^ up to a 200 μM concentration, 10 μM Zn^2+^ had no influence on *Ca*DPP III activity, and no data were available for higher concentration. However, a concentration of Zn^2+^ above a certain limit enabled potent inhibition, e.g., 1 mM and 10 μM Zn^2+^, could completely abolish the enzyme activity of DPP III from *D. melanogaster* and rats. Different sensitivity to various metal ions might be attributed to the coordination bonds with certain residues, inducing the structure changes and the distortion of the active site configuration.

The preferred synthetic substrate Arg_2_-2NA was used to determine the kinetic parameters of *Co*DPP III (Figure 3D). Higher concentrations of substrate could not be measured; therefore, *K*_m_ is well above 100 μM, but could not be determined precisely. The *K*_m_ and *V*_max_ of *Co*DPP III were calculated as 578.91 μM and 0.62 μmol min^−1^ mg^−1^, respectively. The catalytic constant *K*_cat_ and catalytic efficiency *K*_cat_/*K*_m_ were 1.34 s^−1^ and 2.31 mM^−1^ s^−1^, respectively. The *K*_cat_ was three orders of magnitude higher than that of AtND. Nevertheless, the much higher *K*_m_ value for *Co*DPP III resulted in hydrolysis efficiency which was three orders of magnitude lower compared with *Bt*DPP III and *h*DPP III (Table 1). The very high *K*_m_ value, higher than achievable substrate concentrations, suggested Arg_2_-2NA might not be a good substrate for *Co*DPP III.

### 2.3. Overall Structure of CoDPP III

**Figure 5:**
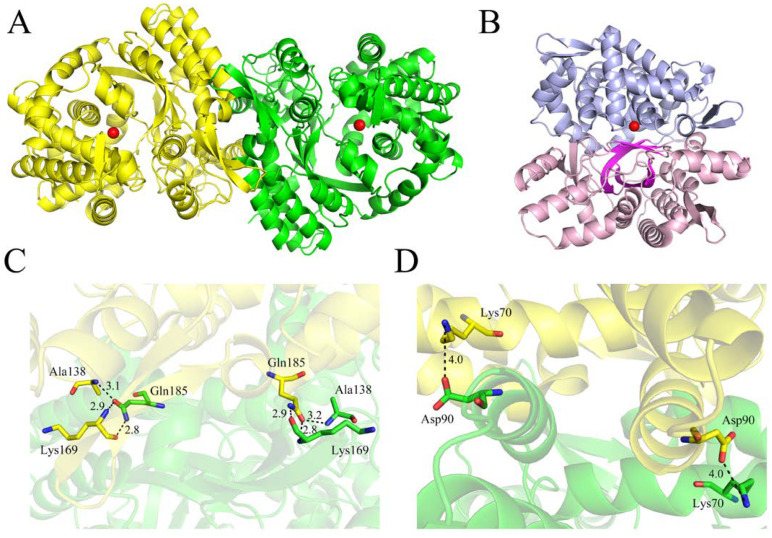
Overview of the structure of *Co*DPP III. (**A**) Ribbon representation of the functional dimer of *Co*DPP III. The two monomers are shown in yellow and green. (**B**) Ribbon representation of *Co*DPP III monomer. The upper and lower domains are shown in light blue and pink, respectively. The five-stranded β-structure is shown in magenta. The catalytic zinc ion is represented by a red sphere. (**C**) Close-up view of the hydrogen bonds formed in the interface. (**D**) Close-up view of the salt bridges formed in the interface.

The crystal structure of *Co*DPP III was refined to a resolution of 1.9 Å (Table 2). There were two almost identical molecules with root-mean-square deviations (rmsd) of 0.23 Å in the asymmetric unit (Figure 5A), indicating the dimer state of *Co*DPP III in solution. The final model was missing 27 amino acids at the N-terminal and 8 and 12 at the C-terminal of chain A and chain B, respectively. It was shown that the monomer structure of *Co*DPP III comprises two domains, which are separated by an inter-domain cleft (Figure 5B). The upper domain encompassing residues (266–303, 345–546) consists of mostly α-helix containing the conserved zinc-binding motifs, and the lower domain encompassing residues (28–265, 304–344, 547–564) contains a five-stranded β-barrel core, which is flanked on one side by α-helix. The dimerization interface area between two molecules was 1416.9 Å^2^, as assessed using PDBePISA. Different from the formation of intermolecular disulfide bonds in *y*DPP III, the interface of *Co*DPP III is formed through intramolecular interactions between the lower domain with two salt bridges, six hydrogen bonds, and ninety-three non-bonded contacts between 27 residues from Chain A and 28 residues from Chain B. Hydrogen bonds are formed between three residues (Ala-138, Lys-169, and Gln-185) (Figure 5C), and salt bridges are formed between the Asp-90 and Lys-70 of each monomer (Figure 5D).

**Figure 6:**
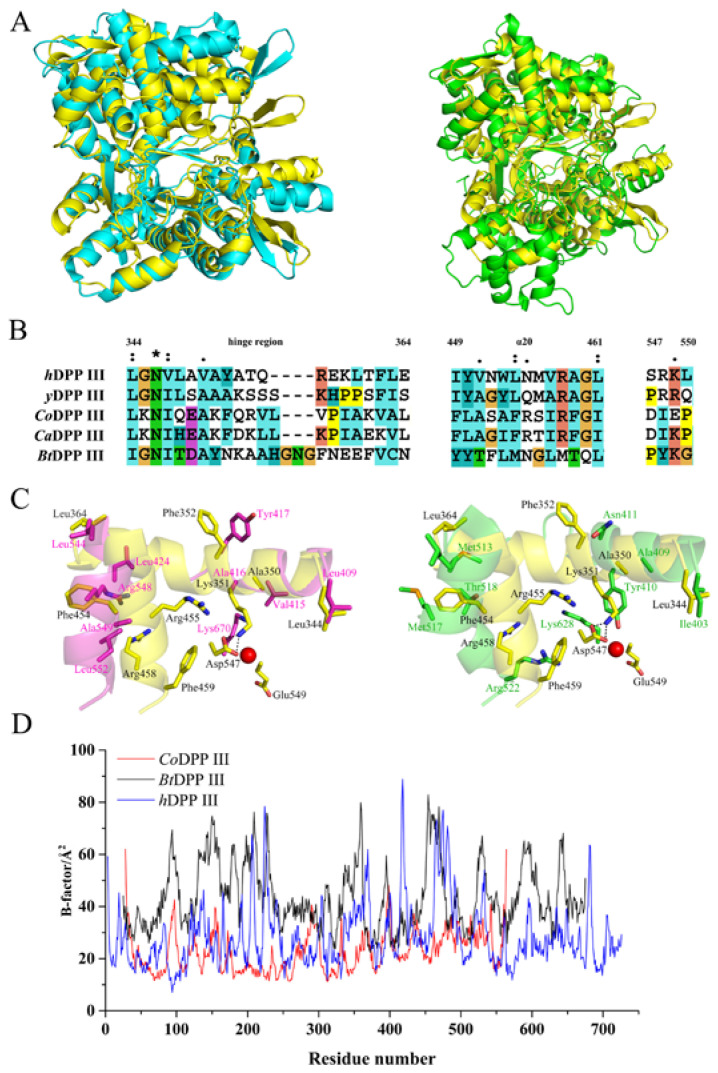
Structure and sequence comparison of *Co*DPP III with other DPP III. (**A**) Structure overlay of *Co*DPP III (yellow) with *Ca*DPP III (cyan, PDB ID: 6EOM) and closed *Bt*DPP III (green, PDB ID: 5NA8). (**B**) Sequence alignment of the hinge region. Amino acid residues in *Co*DPP III are noted above the sequences. (**C**) Structural alignment of the hinge region of *Co*DPP III (yellow) with closed *h*DPP III (magenta, PDB ID: 3T6B) and closed *Bt*DPP III (green, PDB ID: 5NA8). The catalytic zinc ion is represented by a red sphere. (**D**) Residue based B-factors of *Co*DPP III, *Bt*DPP III, and *h*DPP III.

The DALI results indicated that the monomer structure of *Co*DPP III is highly homologous to all other available crystal structures of M49 family enzymes. It is most similar to *Ca*DPP III (PDB: 6EOM, rmsd 3.8 Å for aligned 521 residues), followed by *Bt*DPP III in the closed form (PDB: 5NA8, rmsd 3.0 Å for aligned 500 residues), *h*DPP III in complex with angiotensin-II (PDB: 5E2Q, rmsd 3.6 Å for aligned 497 residues), and *y*DPP III (PDB: 3CSK, rmsd 4.9 Å for aligned 307 residues). Structure superimposition revealed that the distinct difference between *Co*DPP III and *Ca*DPP III was caused by the bending of η2, α6, α18, α23, β9, and β10 in the upper domain and α6, α7, β2, and β3 in the lower domain (Figure 6A). Nevertheless, superposition on the corresponding upper and lower domains in *Co*DPP III and *Ca*DPP III structures yielded rmsd values of 0.863 and 0.678 Å, indicating the closely similar fold of the two separate domains because of the high sequence identity.

All the determined structures and MD simulations showed that DPP III goes through conformation change from open to closed upon ligand binding [25,42,49]. The closed conformation of the unbound enzyme was also obtained with the co-crystallization of *h*DPP III, which is likely induced by the procedure of seeding with microcrystals of the ligand complex [26]. In the case of the partially closed *Ca*DPP III, it was assumed that the Ala-Lys dipeptide in the active site was introduced during protein expression [21,22]. However, the molecular architecture of *Co*DPP III is more compact and resembles the closed conformation, independent of the substrate binding (Figure 6A). Although many attempts have been made, we could not obtain the open structure in the ligand-free state.

The conformational motion of *h*DPP III has been attributed to the rearrangement of the hinge region, which is stabilized by hydrogen bonds by Lys670 in an open conformation and moves outward to the substrate-binding pocket in a closed conformation [23]. The sequence alignment of the hinge region indicated that it is partially conserved between eukaryotic and bacterial DPP III (Figure 6B). The C-terminal part of the hinge region in *h*DPP III (residues 409–429) and *Bt*DPP III (residues 403–423) is a U-shape conformation, whereas the hinge region in *Co*DPP III (residues 344–364) and *Ca*DPP III (residues 340–360) adopts an α-helix conformation (Figure 6C). Early studies proposed that B-factor has been a means to identify flexibility and rigidity in protein; high B-factors and low B-factors are indicative of higher flexibility and rigidity, respectively [50]. A comparison of B-factors in Figure 6D showed that the residues located at the hinge region in *Co*DPP III are more rigid than those in *h*DPP III and *Bt*DPP III. The conserved Lys670 of *h*DPP III and Lys628 is replaced by Glu549 of *Co*DPP III in sequence, but it is structurally equivalent to Asp547, which stabilizes the closed conformation of *Co*DPP III by forming hydrogen bonds to Lys351 in the hinge region (Figure 6C). Moreover, structural alignment showed that α20 of *Co*DPP III is closer to the hinge region, and the steric hindrance of Arg455 and Phe459 may impede the movement of the hinge region into the substrate binding pocket. These sites are amino acids with shorter side-chains in *h*DPP III (Ala549 and Leu552) and *Bt*DPP III (Met517 and Thr518).

### 2.4. Active Site of CoDPP III

**Figure 7:**
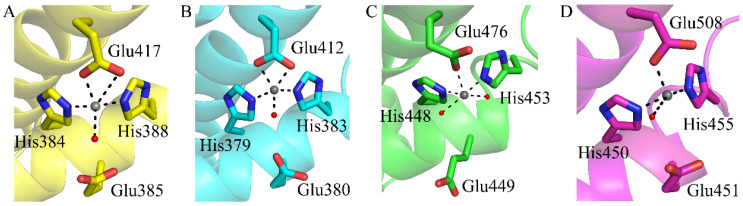
Close-up views of the zinc binding site. (**A**) *Co*DPP III. (**B**) *Ca*DPP III (PDB ID: 6EOM). (**C**) *Bt*DPP III (PDB ID: 5NA7). (**D**) *h*DPP III (PDB ID: 3FVY). The catalytic zinc ion and water are represented by a gray and red sphere, respectively.

Compared to the conserved hexapeptide motif, there is no conspicuous difference in the Zn^2+^ binding position with the diminished pentapeptide motif. The Zn^2+^ coordination in the active site of *Co*DPP III is pentacoordinated by two histidines from the first conserved motif H^384^ELMH^388^, one glutamic acid from the second motif E^417^EAKAD, and a water molecule (Figure 7A). Similarly, a square pyramidal with two water molecules in *Bt*DPP III and bidentated with glutamic acid in *Ca*DPP III was observed (Figure 7B and C), whereas the orientation of glutamic acid from the second motif is distinct in the case of bacterial DPP III, which functions to activate the water molecules for the nucleophilic attack. In contrast, Zn^2+^ is tetracoordinated in the eukaryotic counterparts (Figure 7D). The site-directed mutagenesis also confirmed the key role of the four residues in catalysis for *Co*DPP III.

### 2.5. Mode of Substrate Binding 

**Figure 8:**
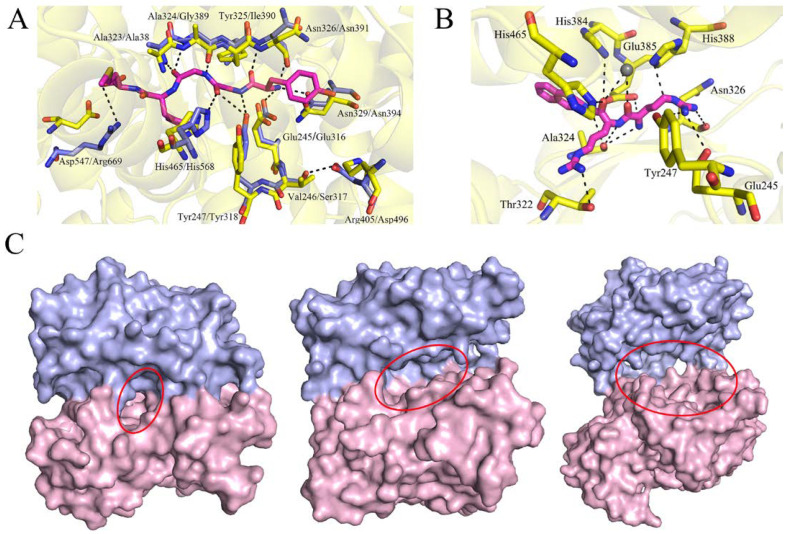
Substrate binding site of *Co*DPP III. (**A**) Structure overlay of *Co*DPP III (yellow) and the substrate binding site of *h*DPP III complex (light blue, PDB ID: 5E33). The hydrogens between the amino acids of *h*DPP III and Met-enkephalin (magenta) are shown as black dash lines. (**B**) Interaction between the docked Arg_2_-2NA (magenta) and the amino acids in the binding site of *Co*DPP III (yellow). The hydrogens are shown as black dash lines. The catalytic zinc ion and water are represented by a gray sphere. (**C**) Surface representations of *Co*DPP III (left), *Ca*DPP III (middle, PDB ID: 6EOM), and *Bt*DPP III (right, PDB ID: 5NA7). The upper and lower domains are shown in light blue and pink. The substrate binding sites are marked by red ellipse.

The substrate binding of DPP III has been best studied in *h*DPP III, including opioid peptides (Met- and Leu-enkephalin, endomorphin-2), peptide inhibitors (tynorphin and aprotinin), and angiotensin-II [26,51,52]. The overall binding mode of the studied peptides were found to be similar, and the N-terminal residues were shown to form extensive interactions with the five-stranded β-core of DPP III in an antiparallel fashion. Therefore, we compared the active sites of *Co*DPP III and *h*DPP III with bound Met-enkephalin (Figure 8A). Most residues hydrogen-bonded with enkephalin in *h*DPP III are structurally conserved in *Co*DPP III. The exceptions are Gly389, Ile390, and Arg669, which correspond to Ala324, Tyr325, and Asp547 in *Co*DPP III, respectively. In addition, Asp496 in *h*DPP III has been identified as an important residue to determine substrate specificity, which is replaced by Arg405 in *Co*DPP III [53]. It is suggested that the H-bonding interaction between Ser317 and Asp496 contributes to the binding of the N-terminal residues of the substrate through the influence of the position of Glu316. Instead of Ser317, a valine is found in the equivalent position in *Co*DPP III.

Despite multiple attempts, we were unable to obtain the complex structure, and Arg_2_-2NA was docked to investigate the effect of ligand binding (Figure 8B). The N-terminus of Arg_2_-2NA is anchored to the enzyme through electrostatic interactions and the hydrogen bonding from the side chains of Glu245, Tyr247, and Asn326, while the side chains of substrate arginine form polar contacts with Thr322, Ala324, His385, and His388. In addition, the carbonyl oxygen of the Arg backbone is stabilized by Zn^2+^, His384, and His465, and naphthalene in the substrate forms a π–π interaction with His384. Although most amino acids involved in the substate binding are also conserved in *Ca*DPP III and *Bt*DPP III, the *K*_m_ value of *Co*DPP III is much higher than that of them. As mentioned above, in contrast to the opening cleft of other characterized DPP III, the ligand-free *Co*DPP III displays the closed conformation, which creates a narrower entrance to the substrate binding site and unfavorable binding of the bulky naphthalene ring (Figure 8C). 

### 2.6. CoDPP III Promotes the Developmental Process of M. xanthus DK1622

**Figure 9:**
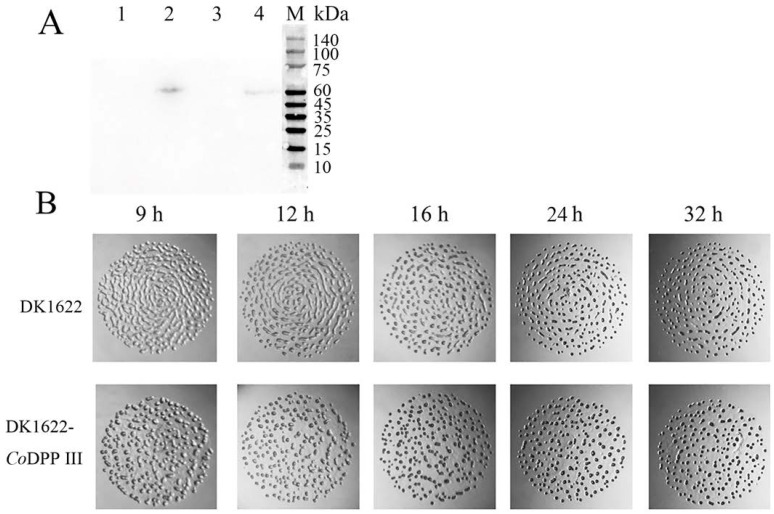
Analysis of *Co*DPP III expression and developmental timing in wild-type and mutant strains. (**A**) Western blot using anti-his antibody. *Co*DPP III was expressed in vegetative cells (lane 2) and developmental cells (lane 4) in DK1622-*Co*DPP III, but not in wild-type DK1622 (lane 1 and 3). (**B**) Developmental time course of fruiting body morphogenesis on TPM starvation agar.

Although several DPP III were cloned and characterized, their physiological significance remains obscure in terms of bacterial function. As genetic manipulation has not been established for strain EGB, we utilized the myxobacteria model strain *Myxococcus xanthus* DK1622 to explore the function of DPP III. Despite the 68% sequence identity of DPP III in *M. xanthus* DK1622 with *Co*DPP III, DPP III expression was not detectable in DK1622 during the vegetative growth and development stage by RT-PCR. The non-expression of endogenous DPP III in *M. xanthus* DK1622 made it served as a model of DPP III deletion mutant for *Corallococcus* sp. EGB. The effects of ectopic expression of *Co*DPP III in *M. xanthus* DK1622 would appropriately elaborate its function in *Corallococcus* sp. EGB. Subsequently, *Codpp III* was inserted into the DK1622 genome via attB-site-directed recombination. Western blot showed that *Co*DPP III was successfully expressed in the vegetative and developmental cells of strain DK1622-*Co*DPP III (Figure 9A), and the phenotype of the mutant was comparable to that of DK1622. However, a discrepancy in the fruiting body morphogenesis was observed (Figure 9B). Normally, *M. xanthus* development progresses in a reproducible manner that starts with the aggregation of cells to form a mound, and finally form the fully mature fruiting bodies filled with myxospores. During the development of the wild-type strain DK1622, elongated ridges were found at 9 h after the initiation of starvation; cells left ripples and migrated into the aggregation center to form mounds within 12–16 h. As starvation continued, the hemispherical mounds composed of packed cells developed into darkened fruiting bodies. Unlike the wild-type strain, the mutant strain completed the aggregation, and the mounds grew in larger size separately after 9 h of starvation. The formation of fruiting bodies of the mutant was originated within 12–16 h, an approximately 12 h head start in development between the 12 and 24 h time points. By 32 h, the mutant displayed more separate and circular symmetric fruiting bodies, which would appear after 48 h of starvation for wild-type DK1622. Meanwhile, the viable spore production of the mutant and wild-type strains was determined as 8.05 × 10^4^ and 6.75 × 10^4^ spores mL^−1^, respectively, a nearly 20% increase in spore viability. The sporulation levels, corroborating the observed advancement in fruiting body morphogenesis, suggested an effect of *Co*DPP III in promoting the developmental process of *M. xanthus*.

### 2.7. A-Signal from DK1622-CoDPP III Restored Development in asgA Mutant

**Figure 10:**
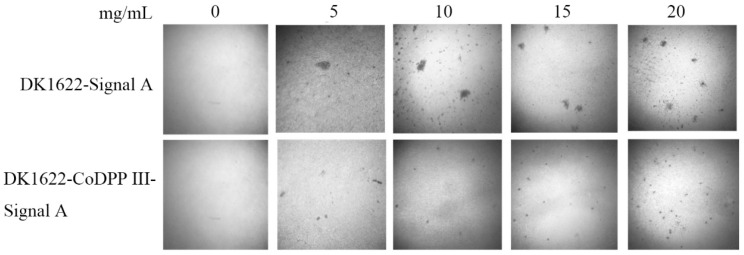
Expression of *Co*DPP III rescues the developmental defects of *asgA* mutant under submerged culture conditions.

Fruiting body development is induced by starvation; five classes of signal have been described as being involved in fruiting body formation and sporulation, named A-, B-, C-, D-, and E-signals [54]. Among the proposed five signals, the heat stable fraction of the A-signal is a mixture of amino acids and peptides [34], which is required for the development after 2 h of starvation. If a sufficiently high concentration of A-signal is reached, the expression of A-signal-dependent genes are regulated by the sensed A-signal, resulting in the initiation of the developmental program [55]. In light of the peptidase activity of *Co*DPP III, we speculated that *Co*DPP III affects developmental timing through the A-signal pathway. Thus, we tested the development in an *asgA* mutant using the A-signal bioassay. In agreement with the earlier observations, our constructed *asgA* mutant was unable to aggregate or sporulate under submerged culture conditions in the absence of the A-signal. The addition of the A-signal that isolated from the starving wild-type cells and *Co*DPP III expression mutant rescued the developmental defect of the *asgA* mutant. As the concentration of the A-signal increased, more induced fruiting body formation was observed, indicating the concentration-dependent effect of the A-signal on the developmental rescue (Figure 10). In comparison, in the case of A-signal addition isolated from the *Co*DPP III expression mutant, efficient sporulation in the rescued fruiting bodies was observed by more numerous and closely packed fruiting bodies (black dots), which is consistent with the abovementioned result regarding the TPM starvation agar. Thus, it was suggested that *Co*DPP III might be involved in the further processing of protein fragments to smaller peptides and amino acids, which acted as the A-signal.

## 3. Materials and Methods

### 3.1. Strains and Cultures

The strains and plasmids used are listed in Table S2. *Corallococcus* sp. strain EGB (CTCC No. M2012528) was cultivated in the medium consisting of 0.5% (*w*/*v*) yeast extract, 1% (*w*/*v*) tryptone, 1% (*w*/*v*) soluble starch, and 0.1% CaCl_2_ (*w*/*v*) on a rotary shaker at 180 rpm at 30 °C for 4 days. The *M. xanthus* DK1622 was cultured in CYE (1.0% Bacto Casitone, 0.5% Difco yeast extract, 10 mM 3-[N-morpholino] propanesulfonic acid [MOPS], pH 7.6, and 0.1% MgSO_4_) broth at 30 °C.

### 3.2. Gene Cloning and Sequence Analysis of CoDPP III

The gel slice containing *Co*DPPIII was analyzed by peptide mass fingerprinting (Boyuan Bio-Tech, Shanghai, China), and the results were analyzed based on searches of the Mascot database (Matrix Science). According to the result of peptide mass fingerprinting and the genome sequence of *C. coralloides* DSM 2259 (GenBank accession no. CP003389.1), the open reading frame encoding *Co*DPP III was amplified by PCR with primers 5′-CATATGCCCAAGTCCAGAAC-3′ and 5′-CTCGAGCTTCGCCGCGGG-3′ using the genomic DNA of strain EGB as template. The underlined nucleotides represent the restriction enzyme sites. The PCR product was cloned into *Nde* I and *Xho* I sites in pET29a (+) vector with C-terminal hexahistidine tag. The recombined plasmid (pET29a-*Codpp III*) was sequenced by the GenScript Biotech Corporation (Nanjing, China), and the correct recombined plasmid was transformed into *E. coli* BL21(DE3).

The sequence alignment was performed using NCBI BLASTP (http://www.ncbi.nlm.nih.gov (accessed on 10 July 2020)). The molecular weight and the isoelectric point (*pI*) were calculated with the ExPASy Proteomics server (https://www.expasy.org (accessed on 10 July 2020)). The putative signal peptide was predicted using the SignalP server (http://www.cbs.dtu.dk/services/SignalP (accessed on 10 July 2020)). The sequence alignment was performed using Clustal W [56], and the result was generated with ESPript [57]. The phylogenetic analysis was conducted using MEGA version 7 [58].

### 3.3. Protein Expression and Purification of CoDPP III

*E. coli* BL21(DE3) carrying pET29a-*Codpp III* were grown in LB medium containing 50 mg/L kanamycin at 37 °C until the *OD*_600_ reached 0.6. The protein expression was induced with 0.2 mM isopropyl-β-D-thiogalactopyranoside (IPTG) by incubation at 16 °C for 24 h. The cells were harvested (12,000 rpm, 20 min, and 4 °C) and resuspended in 50 mM sodium phosphate buffer (pH 7.0). The suspended solids were then disrupted by ultrasonic disruption, and the lysates were centrifuged to obtain the supernatant. The supernatant was loaded on CM-Sepharose fast flow pre-equilibrated with 20 mM sodium phosphate buffer (pH 6.0). Unbound proteins were eluted from the column with the same buffer. The bound proteins were eluted with the equilibrated buffer containing 0.25 M NaCl. The eluted enzyme was pooled and dialyzed against 20 mM Tris-HCl (pH 7.0). The protein concentration was determined according to the method of Bradford using bovine serum albumin as the standard [59]. The purified enzyme was concentrated to 10 mg/mL by ultrafiltration for crystallization.

Selenomethionine (Se-Met) labeled DPP III was overexpressed using *Escherichia coli* B834(DE3) cells transformed with the previously described construct. The transformed colony was inoculated into LB medium containing 50 mg/L kanamycin and was grown at 37 °C for 12 h. The cell culture was collected and washed twice using sterile H_2_O, and then, it was added into M9 medium (22 mM Na_2_HPO_4_, 22 mM KH_2_PO_4_, 18 mM NH_4_Cl, and 12.5 mM NaCl) supplemented with 60 mg/L L-methionine, 2 mM MgSO_4_, 2 mg/L biotin, 0.5% glucose, 2 mg/L thiamine, and 50 mg/L kanamycin. The cells were grown overnight at 37 °C. The cell culture was then collected, washed, and inoculated as described above, with the exception that L-methionine was absent from the M9 medium. The cells were grown at 28 °C and 180 rpm until the *OD*_600_ reached a constant value, and 60 mg/L Se-Met was added to the cell culture. The cells were grown at 16 °C to an *OD*_600_ of approximately 0.5, followed by induction with 0.4 mM IPTG at 16 °C for 24 h. The purification of Se-Met labeled DPP III was accomplished according to the same protocol as for the native DPP III.

### 3.4. Determination of Molecular Mass

The homogeneity and subunit molecular mass of *Co*DPP III were estimated using 12% SDS-PAGE gels. The native molecular mass of *Co*DPP III was determined using gel filtration chromatography with a HiLoad 16/600 Superdex 200 pg column (GE Healthcare, Chicago, IL, USA) in 20 mM Tris-HCl (pH 7.0) containing 0.1 M NaCl at a flow-rate of 1 mL/min. Ovalbumin (44 kDa), conalbumin (75 kDa), aldolase (158 kDa), and ferritin (440 kDa) were used as molecular mass standards.

### 3.5. Biochemical Characterization of CoDPP III

The enzymatic activity was assayed by 30 min incubation at 50 °C with 15 μg of purified *Co*DPP III and 40 μM of Arg-Arg-2-naphthylamide (Arg_2_-2NA) as substrate in 20 mM pH 7.0 Tris-HCl buffer, in a total volume of reaction mixture of 1 mL, using the colorimetric method as described previously [29].

The optimal temperature for *Co*DPP III was determined at temperatures from 30 °C to 70 °C by the standard assay with the addition of 0.1 mM CoCl_2_. The thermal stability was assayed using the residual enzyme activity after the pre-incubation of *Co*DPP III at different temperatures (30–70 °C) for 1 h. The pH optimum was estimated in 20 mM citrate buffer (pH 3.0–6.0), sodium-phosphate buffer (pH 6.0–7.0), Tris-HCl buffer (pH 7.0–9.0), and Glycine-NaOH buffer (pH 9.0–10.0). The enzyme activity was calculated as a percent of maximum activity.

The effect of metal ions on enzyme activity was investigated with native and chelating agent treated enzymes. *Co*DPP III was incubated with 1 mM EDTA at 50 °C for 1 h and dialyzed at 4 °C overnight against 20 mM pH 7.0 Tris-HCl; then, the dialyzed enzyme was assayed using the standard procedure with the addition of 0.1 mM, 0.5 mM, and 1mM chloride salt of different ions, respectively. The native enzyme activity was measured in the presence of 0.001–0.5 mM various metal ions under the standard assay condition. The residual enzyme activity was calculated as a percent of activity as compared to the control.

The kinetic parameters (*K*_m_ and *V*_max_) for the hydrolysis of Arg_2_-2NA were determined at 50 and pH 7.0 containing 0.1 mM CoCl_2_. The kinetic parameters were calculated using non-linear fitting to the Michaelis–Menten curve (Origin 9.0), with substrate concentrations in the range of 10–120 μM.

### 3.6. Crystallization, Data Collection, Structure Determination, and Refinement

Crystallization was performed using the sitting-drop vapor-diffusion method by mixing 1 μL of protein solution at 8.0 mg/mL and 1 μL of crystallization solution at 20 °C. Initial crystals were found in a condition containing 0.1 M sodium acetate trihydrate, pH 4.5, and 25% *w*/*v* PEG 3350. After a systematic grid screening of protein concentrations, pH, and precipitant concentrations, further optimization was conducted using the commercial additive screening kit (Hampton Research). Improved crystals were produced in the condition containing 0.1 M sodium acetate trihydrate, pH 4.59, 23.5% *w*/*v* PEG 3350, and 0.1% n-Octyl-β-D-glucoside using protein concentrated to 2.2 mg/mL. On the basis of the crystal condition of wild protein, crystals of Se-Met-labelled protein were obtained in 0.1 M sodium acetate trihydrate, pH 4.59, 24% *w*/*v* PEG 3350, and 0.01 M TCEP hydrochloride using protein concentrated to 5.45 mg/mL.

The crystals were flash-cooled in liquid nitrogen in mother liquor containing 20% (*v*/*v*) glycerol as a cryoprotectant, and the X-ray diffraction data sets were collected at 100 K at beamline BL17U1 at the Shanghai Synchrotron Radiation Facility. All the X-ray diffraction data were processed using XDS [60]. The single-wavelength anomalous diffraction (SAD) experiment with Se-Met-labelled crystal was performed at 0.9791 Å wavelength. Heavy atom sites were found with SHELXC/D. Phases were calculated and extended to 1.90 Å resolution using OASIS [61]. The structure model was manually adjusted with Coot [62] and refined with Refmac5 [63]. Details of the data collection, processing, and structure refinement are summarized in Table 2. The B-factors were calculated using the temperature factor analysis in CCP4. All figures were prepared using PyMOL [64]. The coordinates and the related structural factors were deposited in the Protein Data Bank (PDB) under the code number 5ZUM.

### 3.7. Molecular Docking

The molecular structure of Arg_2_-2NA was drawn with ChemDraw 21.0.0 and energy minimized using Molecular Operating Environment (MOE 2019). The structure of *Co*DPP III was configured through the deletion of water molecules and the addition of polar hydrogens. The docking of Arg_2_-2NA was performed with the default docking protocol implanted in MOE software. The initial scoring was set at London dG and rescoring was set at GBVI/WSA dG. Based on the protein–ligand interaction, the top-ranking docked pose was selected for the analysis of the binding mode.

### 3.8. RT-PCR

Total RNA was isolated from *M. xanthus* DK1622 cells during vegetative growth and development for 9 h and 24 h using an RNA purification Kit (PuDi Biotech, Shanghai, China), and it was treated with DNase I to remove residual DNA. The reverse primer of the primer pair was used as the primer to generate cDNA using total RNA as the template. The cDNA was verified with a fragment of the *Oar* gene (264 bp) as a positive control. The PCR fragment of DPP III was amplified with the forward primer GGTGGAGGCGTGGGTGAAGT and the reverse primer CGCTGGCGTAGTAGTCGTTGG. The size of the PCR product was expected to be 247 bp.

### 3.9. Heterologous Expression of Codpp III in M. xanthus DK1622

A 2697 bp fragment containing the *Codpp III* gene and its promoter was amplified using EGB genomic DNA as the template with primer pair 5′-CCCAAGCTTTGAACTGCTGCGTGCCG-3′ and 5′-CCGCTCGAGCTACTTCGCCGCGGACTTC-3′. The product was digested with *Hind* III and *Xho* I, then ligated with pET29a-*attP*, resulting pET29a-*attP*-*Codpp III* [65]. DK1622-*Codpp III* was constructed via the integration of the plasmid into the *attB* site through electroporation into DK1622 as previously described [66]. The *M. xanthus* DK1622 was cultured in 50 mL CYE medium until the optical density at 600 nm (*OD*_600_) was 0.6. Then, 8 mL of the culture was collected by centrifugation (8000 rpm, 5 min) at 4 °C, washed thrice with sterile water, and resuspended with 400 μL of sterile water. A 40 μL aliquot of competent cells was electroporated with 5 μL of plasmid DNA in a 1 mm cuvette (650 V, 400 Ω, 25 mF). The cells were grown in 2 mL of CYE medium in a 50 mL tube (30 °C, 200 rpm and 4 h). After recovery, 500 μL of cells was mixed with soft agar and poured onto a CYE agar plate which was supplemented with 40 μg/mL kanamycin. The plates were incubated at 30 °C and transformants could typically be observed 5–7 days after electroporation. The integrated transformants were confirmed by PCR using primers binding to *Codpp III*. 

### 3.10. Western Blot Analysis

The cell lysates of DK1622 and DK1622-*Codpp III* were prepared from cells during vegetative growth and 24 h of development. Proteins were separated by 12% SDS-PAGE and electroblotted onto PVDF membrane (Millipore). Each set was probed with a 1:1000 dilution of primary antibody composed of mouse anti-His tag monoclonal antibody (Zoonbio Biotechnology, Nanjing, China), followed by incubation with the secondary antibody horseradish peroxidase conjugated goat anti-mouse IgG at a dilution of 1:10000 (Zoonbio Biotechnology, China). The immunoblot was developed with the BeyoECL Plus detection kit (Beyotime Biotechnology, China) according to the manufacturer’s instructions.

### 3.11. Construction of asgA Mutant

For *asgA* disruption, an internal 1073 bp fragment of *asgA* was amplified using DK1622 genomic DNA as a template with the primer pair 5′-CCCAAGCTTCCTCCGGGTCTTCGACGCGAACTTC-3′ and 5′-CCGGAATTCCGATTTCCGTGCCCTCCCCTTGCGT-3′, and it was then cloned into *Hind* III and *EcoR* I sites of pBJ113, generating pBJ113-*asgA*. The constructed plasmids were electroporated into the wild-type strain DK1622. The mutated strains were selected on a CYE agar plate with 40 μg/mL kanamycin and verified by PCR analysis with primer pair 5′-TGCGGTACCCAGCAGATCGCCAAGG-3′ and 5′-GGCGGTGGTGCCCTTCGACGCG GTG-3′, located upstream and downstream on the chromosome from the *asgA*.

### 3.12. Isolation of the A-Signal

A-signal production was carried out as previously described [34]. The *M. xanthus* DK1622 and DK1622-*Co*DPP III were cultured in 1 L of CYE broth to an *OD*_600_ of 1.0. The cells were pelleted by centrifugation, washed three times with sterile water, and resuspended to an *OD*_600_ of 10 in MC7 buffer (10 mM morpholine-propanesulfonic acid, 1 mM CaC1_2_, and pH 7.0). This cell suspension was grown at 30 °C for 3 h, and the crude A-signal was harvested by removing the cells. Then, the crude A-signal was heated at 100 °C for 10 min, and the heat stable A-signal was collected by centrifugation. The freeze-dried supernatant was resuspended in deionized water and filtered through a 0.22 mm sterile filter for development assay.

### 3.13. Development Assay

Development was assayed on TPM (10 mM Tris-HCl, pH7.6, 1mM KPO_4_, pH7.6, and 8 mM MgCl_2_) agar plates or in submerged culture as described previously [55]. Briefly, cells were grown in CYE broth to an *OD*_600_ of 1.0, harvested and resuspended in TPM buffer to an *OD*_600_ of 10. For development on TPM agar, 10 µL of cell suspension was spotted and incubated for the indicated periods of time at 30 °C. For the A-signal bioassay under the submerged starvation condition, the prepared A-signal was added to the cell suspension at a final concentration of 0–20 mg/mL. Aliquots of 400 µL were transferred to wells of a 24-well microtiter plate and incubated at 30 °C for 24 h. Fruiting body formation was visually recorded using a Nikon stereomicroscope.

### 3.14. Sporulation Assay

Spore viability was assayed as described in [67]. In the present study, cells were grown in CYE broth, harvested, and resuspended to an *OD*_600_ of 10 in TPM buffer. A 300 µL cell suspension was spotted on TPM plates and incubated at 30 °C. The cells at different developmental periods were collected and resuspended in 2 mL of ddH_2_O. The fruiting bodies were sonicated at an interval of 9.9 s three times and then heated at 50 °C for 2 h. Spores were diluted, and 100 µL serial dilutions were added to 4 mL CYE agar plates. The plates were incubated at 30 °C for 4 days before the colonies were counted.

## 4. Conclusions

This is the first report to outline dipeptidyl peptidase III distribution in myxobacteria; we characterized a new type of DPP III in *Corallococcus* sp. strain EGB. The sequence analysis revealed that *Co*DPP III contains a pentapeptide HEXXH active-site zinc-binding motif, consisting of an atypical subgroup of the peptidase family M49 together with *Ca*DPP III. Different from the open and partially closed structures, *Co*DPP III displayed a closed conformation without substrate binding, expanding our knowledge of DPP III’s conformation. Combining the structure comparison with B-factor analysis, the higher rigidity of the helix in the hinge region and its steric conflicts with α20 provided an explanation for the behavior of protein closure. Correspondingly, the relatively small inter-domain cleft created a narrower entrance to the substrate binding site and led to the decrease in the enzyme activity. In addition, the function of *Co*DPP III involved in the fruiting body development of myxobacteria was firstly elucidated. According to the current model for the mechanism of the A-signal, *Co*DPP III hydrolyzed the peptides of its optimum substrate length, causing the release of peptides that act as A-signal activity. 

## Figures and Tables

**Table 1:** Kinetic parameters of the reported DPP III.

	*K*_m_/μM	*K*_cat_/s^−1^	*K*_cat_/*K*_m_/mM^−1^ s^−1^	Reference
AtND	3.25 ± 1.12	0.002 ± 0.0001	0.615	[16]
PpND	10.63 ± 1.72	0.014 ± 0.003	1.289	[16]
*h*DPP III	2.2 ± 0.1	20.5 ± 1.2	9318.2	[48]
*y*DPP III	12.0 ± 1.7	0.18 ± 0.06	15.0	[48]
*Bt*DPP III	2.5 ± 0.9	5.0 ± 2.9	2000	[29]
*Pg*DPP III	0.97 ± 0.16	0.75 ± 0.13	773.2	[15]
*Ca*DPP III	35.2 ± 2.0	3.07 ± 0.04	87.3	[21]
*Pa*DPP III	9.0	0.0292	3.2	[30]
*Co*DPP III	578.91 ± 60.52	1.34 ± 0.06	2.31	

**Table 2:** Data collection and refinement statistics ^a^.

	Se-*Co*DPPIII
**Data collection**	
Space group	*P*2_1_2_1_2_1_
Resolution [Å]	25.0–1.90 (1.97–1.90)
Unit-cell	
*a*/*b*/*c* [Å]	58.14/77.85/228.29
*α*/*β*/*γ* (°)	90.00/90.00/90.00
Unique reflections	82,558 (7990)
Redundancy	4.3 (4.3)
Completeness [%]	99.7 (98.2)
average *I*/*σ*(*I*)	24.2 (9.6)
R_merge_ [%]	6.7 (20.2)
**Refinement**	
Number of molecules in a.u.	2
No. of reflections	78,400 (5879)
R_work_ (95% of data)	0.173 (0.210)
R_free_ (5% of data)	0.223 (0.260)
R.m.s.d. bonds [Å]	0.010
R.m.s.d. angles [º]	1.515
No. of non-H atoms/average B-factors [Å^2^]	
Protein	8302/24.74
Water	934/31.81
Ligand	2/29.34
**Ramachandran plot**	
Most favored [%]	97.56
Allowed [%]	2.35
Disallowed [%]	0.09

^a^ Values in parentheses are for the highest resolution shell.

## Data Availability

Atomic coordinates and structure factors have been deposited in the Protein Data Bank (PDB) under accession code 5ZUM.

## Supplementary Materials

The following supporting information can be downloaded at: https://www.mdpi.com/article/10.3390/ijms24010631/s1.

## References

[1] Prajapati S.C., Chauhan S.S. (2011). Dipeptidyl peptidase III: A multifaceted oligopeptide N-end cutter. FEBS J..

[2] Simaga S., Babić D., Osmak M., Sprem M., Abramić M. (2003). Tumor cytosol dipeptidyl peptidase III activity is increased with histological aggressiveness of ovarian primary carcinomas. Gynecol. Oncol..

[3] Jha S., Taschler U., Domenig O., Poglitsch M., Bourgeois B., Pollheimer M., Pusch L.M., Malovan G., Frank S., Madl T. (2020). Dipeptidyl peptidase 3 modulates the renin-angiotensin system in mice. J. Biol. Chem..

[4] Komeno M., Pang X., Shimizu A., Molla M.R., Yasuda-Yamahara M., Kume S., Rahman N.I.A., Soh J.E.C., Nguyen L.K.C., Ahmat Amin M.K.B. (2021). Cardio- and reno-protective effects of dipeptidyl peptidase III in diabetic mice. J. Biol. Chem..

[5] Pang X., Shimizu A., Kurita S., Zankov D.P., Takeuchi K., Yasuda-Yamahara M., Kume S., Ishida T., Ogita H. (2016). Novel Therapeutic Role for Dipeptidyl Peptidase III in the Treatment of Hypertension. Hypertension.

[6] Matić S., Tomašić Paić A., Sobočanec S., Pinterić M., Pipalović G., Martinčić M., Matovina M., Tomić S. (2022). Interdisciplinary Study of the Effects of Dipeptidyl-Peptidase III Cancer Mutations on the KEAP1-NRF2 Signaling Pathway. Int. J. Mol. Sci..

[7] Shukla A.A., Jain M., Chauhan S.S. (2010). Ets-1/Elk-1 is a critical mediator of dipeptidyl-peptidase III transcription in human glioblastoma cells. FEBS J..

[8] Karačić Z., Šupljika F., Tomić A., Brkljačić L., Paić A.T., Ćehić M., Tomić S. (2022). Neuropeptides, substrates and inhibitors of human dipeptidyl peptidase III, experimental and computational study—A new substrate identified. Int. J. Biol. Macromol..

[9] Abramić M., Agić D. (2022). Survey of Dipeptidyl Peptidase III Inhibitors: From Small Molecules of Microbial or Synthetic Origin to Aprotinin. Molecules.

[10] Agić D., Karnaš M., Šubarić D., Lončarić M., Tomić S., Karačić Z., Bešlo D., Rastija V., Molnar M., Popović B.M. (2021). Coumarin Derivatives Act as Novel Inhibitors of Human Dipeptidyl Peptidase III: Combined In Vitro and In Silico Study. Pharmaceuticals.

[11] Hashimoto J., Yamamoto Y., Kurosawa H., Nishimura K., Hazato T. (2000). Identification of dipeptidyl peptidase III in human neutrophils. Biochem. Biophys. Res. Commun..

[12] Hirose J., Iwamoto H., Nagao I., Enmyo K., Sugao H., Kanemitu N., Ikeda K., Takeda M., Inoue M., Ikeda T. (2001). Characterization of the metal-substituted dipeptidyl peptidase III (rat liver). Biochemistry.

[13] Mazzocco C., Gillibert-Duplantier J., Neaud V., Fukasawa K.M., Claverol S., Bonneu M., Puiroux J. (2006). Identification and characterization of two dipeptidyl-peptidase III isoforms in Drosophila melanogaster. FEBS J..

[14] Dhanda S., Singh H., Singh J., Singh T.P. (2008). Functional characterization and specific effects of various peptides on enzymatic activity of a DPP-III homologue from goat brain. J. Enzym. Inhib. Med. Chem..

[15] Hromić-Jahjefendić A., Jajčanin Jozić N., Kazazić S., Grabar Branilović M., Karačić Z., Schrittwieser J.H., Das K.M.P., Tomin M., Oberer M., Gruber K. (2017). A novel Porphyromonas gingivalis enzyme: An atypical dipeptidyl peptidase III with an ARM repeat domain. PLoS ONE.

[16] Karačić Z., Vukelić B., Ho G.H., Jozić I., Sučec I., Salopek-Sondi B., Kozlović M., Brenner S.E., Ludwig-Müller J., Abramić M. (2017). A novel plant enzyme with dual activity: An atypical Nudix hydrolase and a dipeptidyl peptidase III. Biol. Chem..

[17] Nelson K.E., Fleischmann R.D., DeBoy R.T., Paulsen I.T., Fouts D.E., Eisen J.A., Daugherty S.C., Dodson R.J., Durkin A.S., Gwinn M. (2003). Complete genome sequence of the oral pathogenic Bacterium porphyromonas gingivalis strain W83. J. Bacteriol..

[18] Karačić Z., Ban Ž., Macheroux P.J.C.T.i.P., Research P. (2017). A novel member of the dipeptidyl peptidase III family from *Armillariella tabescens*. Curr. Top. Pept. Protein Res..

[19] de Vries L.E., Sanchez M.I., Groborz K., Kuppens L., Poreba M., Lehmann C., Nevins N., Withers-Martinez C., Hirst D.J., Yuan F. (2019). Characterization of *P. falciparum* dipeptidyl aminopeptidase 3 specificity identifies differences in amino acid preferences between peptide-based substrates and covalent inhibitors. FEBS J..

[20] Rawlings N.D., Barrett A.J., Thomas P.D., Huang X., Bateman A., Finn R.D. (2018). The MEROPS database of proteolytic enzymes, their substrates and inhibitors in 2017 and a comparison with peptidases in the PANTHER database. Nucleic Acids Res..

[21] Sabljić I., Tomin M., Matovina M., Sučec I., Tomašić Paić A., Tomić A., Abramić M., Tomić S. (2018). The first dipeptidyl peptidase III from a thermophile: Structural basis for thermal stability and reduced activity. PLoS ONE.

[22] Baral P.K., Jajcanin-Jozić N., Deller S., Macheroux P., Abramić M., Gruber K. (2008). The first structure of dipeptidyl-peptidase III provides insight into the catalytic mechanism and mode of substrate binding. J. Biol. Chem..

[23] Bezerra G.A., Dobrovetsky E., Viertlmayr R., Dong A., Binter A., Abramic M., Macheroux P., Dhe-Paganon S., Gruber K. (2012). Entropy-driven binding of opioid peptides induces a large domain motion in human dipeptidyl peptidase III. Proc. Natl. Acad. Sci. USA.

[24] Sabljić I., Meštrović N., Vukelić B., Macheroux P., Gruber K., Luić M., Abramić M. (2017). Crystal structure of dipeptidyl peptidase III from the human gut symbiont Bacteroides thetaiotaomicron. PLoS ONE.

[25] Tomić A., González M., Tomić S. (2012). The large scale conformational change of the human DPP III-substrate prefers the “closed” form. J. Chem. Inf. Model..

[26] Kumar P., Reithofer V., Reisinger M., Wallner S., Pavkov-Keller T., Macheroux P., Gruber K. (2016). Substrate complexes of human dipeptidyl peptidase III reveal the mechanism of enzyme inhibition. Sci. Rep..

[27] Agić D., Brkić H., Kazazić S., Tomić A., Abramić M. (2019). Aprotinin interacts with substrate-binding site of human dipeptidyl peptidase III. J. Biomol. Struct. Dyn..

[28] Matthews B.W. (1988). Structural basis of the action of thermolysin and related zinc peptidases. Acc. Chem. Res..

[29] Vukelić B., Salopek-Sondi B., Špoljarić J., Sabljić I., Meštrović N., Agić D., Abramić M. (2012). Reactive cysteine in the active-site motif of Bacteroides thetaiotaomicron dipeptidyl peptidase III is a regulatory residue for enzyme activity. Biol. Chem..

[30] Attri P., Jodha D., Singh J., Dhanda S. (2018). Purification, kinetic and functional characterization of membrane bound dipeptidyl peptidase-III from NCDC 252: A probiotic lactic acid bacteria. Mol. Biol. Rep..

[31] Ye X., Li Z., Luo X., Wang W., Li Y., Li R., Zhang B., Qiao Y., Zhou J., Fan J. (2020). A predatory myxobacterium controls cucumber Fusarium wilt by regulating the soil microbial community. Microbiome.

[32] Whitworth D.E. (2022). Myxobacteria: Physiology and Regulation. Microorganisms.

[33] Bretl D.J., Kirby J.R. (2016). Molecular Mechanisms of Signaling in Myxococcus xanthus Development. J. Mol. Biol..

[34] Kuspa A., Plamann L., Kaiser D. (1992). Identification of heat-stable A-factor from Myxococcus xanthus. J. Bacteriol..

[35] Ensign J.C., Wolfe R.S. (1965). Lysis of Bacterial Cell Walls by an Enzyme Isolated from a Myxobacter. J. Bacteriol..

[36] Ensign J.C., Wolfe R.S. (1966). Characterization of a small proteolytic enzyme which lyses bacterial cell walls. J. Bacteriol..

[37] Wingard M., Matsueda G., Wolfe R.S. (1972). Myxobacter AL-1 protease II: Specific peptide bond cleavage on the amino side of lysine. J. Bacteriol..

[38] Gnosspelius G. (1978). Purification and properties of an extracellular protease from Myxococcus virescens. J. Bacteriol..

[39] Petit F., Guespin-Michel J.F. (1992). Production of an extracellular milk-clotting activity during development in Myxococcus xanthus. J. Bacteriol..

[40] Poza M., Sieiro C., Carreira L., Barros-Velázquez J., Villa T.G. (2003). Production and characterization of the milk-clotting protease of Myxococcus xanthus strain 422. J. Ind. Microbiol. Biotechnol..

[41] Li Z., Wu J., Zhang B., Wang F., Ye X., Huang Y., Huang Q., Cui Z. (2015). AmyM, a Novel Maltohexaose-Forming α-Amylase from Corallococcus sp. strain EGB. Appl. Environ. Microbiol..

[42] Jajcanin-Jozić N., Deller S., Pavkov T., Macheroux P., Abramić M. (2010). Identification of the reactive cysteine residues in yeast dipeptidyl peptidase III. Biochimie.

[43] Lynn K.R. (1991). The isolation and some properties of dipeptidyl peptidases II and III from porcine spleen. Int. J. Biochem..

[44] Jodha D., Attri P., Khaket T.P., Singh J. (2013). Isolation, purification and biochemical characterization of dipeptidyl peptidase-III from germinated Vigna radiata seeds. Process Biochem..

[45] Spoljarić J., Salopek-Sondi B., Makarević J., Vukelić B., Agić D., Simaga S., Jajcanin-Jozić N., Abramić M. (2009). Absolutely conserved tryptophan in M49 family of peptidases contributes to catalysis and binding of competitive inhibitors. Bioorg. Chem..

[46] Xu T., Xie C., Yao D., Zhou C.Z., Liu J. (2017). Crystal structures of Aflatoxin-oxidase from Armillariella tabescens reveal a dual activity enzyme. Biochem. Biophys. Res. Commun..

[47] Abramić M., Schleuder D., Dolovcak L., Schröder W., Strupat K., Sagi D., Peter-Katalini J., Vitale L. (2000). Human and rat dipeptidyl peptidase III: Biochemical and mass spectrometric arguments for similarities and differences. Biol. Chem..

[48] Jajčanin-Jozić N., Abramić M. (2013). Hydrolysis of dipeptide derivatives reveals the diversity in the M49 family. Biol. Chem..

[49] Tomin M., Tomić S. (2017). Dynamic properties of dipeptidyl peptidase III from Bacteroides thetaiotaomicron and the structural basis for its substrate specificity—A computational study. Mol. Biosyst..

[50] Sun Z., Liu Q., Qu G., Feng Y., Reetz M.T. (2019). Utility of B-Factors in Protein Science: Interpreting Rigidity, Flexibility, and Internal Motion and Engineering Thermostability. Chem. Rev..

[51] Tomić A., Abramić M., Spoljarić J., Agić D., Smith D.M., Tomić S. (2011). Human dipeptidyl peptidase III: Insights into ligand binding from a combined experimental and computational approach. J. Mol. Recognit. JMR.

[52] Tomić A., Tomić S. (2014). Hunting the human DPP III active conformation: Combined thermodynamic and QM/MM calculations. Dalton Trans..

[53] Abramić M., Karačić Z., Šemanjski M., Vukelić B., Jajčanin-Jozić N. (2015). Aspartate 496 from the subsite S2 drives specificity of human dipeptidyl peptidase III. Biol. Chem..

[54] Kaiser D. (2004). Signaling in myxobacteria. Annu. Rev. Microbiol..

[55] Konovalova A., Wegener-Feldbrügge S., Søgaard-Andersen L. (2012). Two intercellular signals required for fruiting body formation in Myxococcus xanthus act sequentially but non-hierarchically. Mol. Microbiol..

[56] Larkin M.A., Blackshields G., Brown N.P., Chenna R., McGettigan P.A., McWilliam H., Valentin F., Wallace I.M., Wilm A., Lopez R. (2007). Clustal W and Clustal X version 2.0. Bioinformatics.

[57] Robert X., Gouet P. (2014). Deciphering key features in protein structures with the new ENDscript server. Nucleic Acids Res..

[58] Kumar S., Stecher G., Tamura K. (2016). MEGA7: Molecular Evolutionary Genetics Analysis Version 7.0 for Bigger Datasets. Mol. Biol. Evol..

[59] Kielkopf C.L., Bauer W., Urbatsch I.L. (2020). Bradford Assay for Determining Protein Concentration. Cold Spring Harbor Protoc..

[60] Lima F.A., Saleta M.E., Pagliuca R.J., Eleotério M.A., Reis R.D., Fonseca Júnior J., Meyer B., Bittar E.M., Souza-Neto N.M., Granado E. (2016). XDS: A flexible beamline for X-ray diffraction and spectroscopy at the Brazilian synchrotron. J. Synchrotron Radiat..

[61] McCoy A.J., Grosse-Kunstleve R.W., Adams P.D., Winn M.D., Storoni L.C., Read R.J. (2007). Phaser crystallographic software. J. Appl. Crystallogr..

[62] Emsley P., Cowtan K. (2004). Coot: Model-building tools for molecular graphics. Acta Crystallogr. Sect. D Biol. Crystallogr..

[63] Vagin A.A., Steiner R.A., Lebedev A.A., Potterton L., McNicholas S., Long F., Murshudov G.N. (2004). REFMAC5 dictionary: Organization of prior chemical knowledge and guidelines for its use. Acta Crystallogr. Sect. D Biol. Crystallogr..

[64] (2015). The PyMOL Molecular Graphics System.

[65] Leng X., Zhu W., Jin J., Mao X. (2011). Evidence that a chaperone-usher-like pathway of Myxococcus xanthus functions in spore coat formation. Microbiology.

[66] Zhu L.P., Yue X.J., Han K., Li Z.F., Zheng L.S., Yi X.N., Wang H.L., Zhang Y.M., Li Y.Z. (2015). Allopatric integrations selectively change host transcriptomes, leading to varied expression efficiencies of exotic genes in Myxococcus xanthus. Microb. Cell Fact..

[67] Lee B., Schramm A., Jagadeesan S., Higgs P.I. (2010). Two-component systems and regulation of developmental progression in Myxococcus xanthus. Methods Enzymol..

