# Supplementary material for: Structural and Functional Characterization of a New Bacterial Dipeptidyl Peptidase III Involved in Fruiting Body Formation in Myxobacteria
Source: Int J Mol Sci. 2022 Dec 30;24(1):631. doi: 10.3390/ijms24010631 (PMC9820243; doi:10.3390/ijms24010631)
Supplement: Supplementary file 1 [file ijms-24-00631-s001.zip › ijms-2032898-supplementary.pdf]

Structural and Functional Characterization of a New Bacterial Dipeptidyl Peptidase III Involved in Fruiting Body Formation in Myxobacteria

Si-Bo Chen [1], Han Zhang [1], Si Chen [1], Xian-Feng Ye [1], Zhou-Kun Li [1], Wei-Dong Liu [2], Zhong-Li Cui [1] and Yan Huang [1,*]

[1] Key Laboratory of Agricultural Environmental Microbiology, Ministry of Agriculture and Rural Affairs, College of Life Sciences, Nanjing Agricultural University, Nanjing 210095, China

[2] Industrial Enzymes National Engineering Laboratory, Tianjin Institute of Industrial Biotechnology, Chinese Academy of Sciences, Tianjin 300308, China

* Correspondence: huangyan@njau.edu.cn

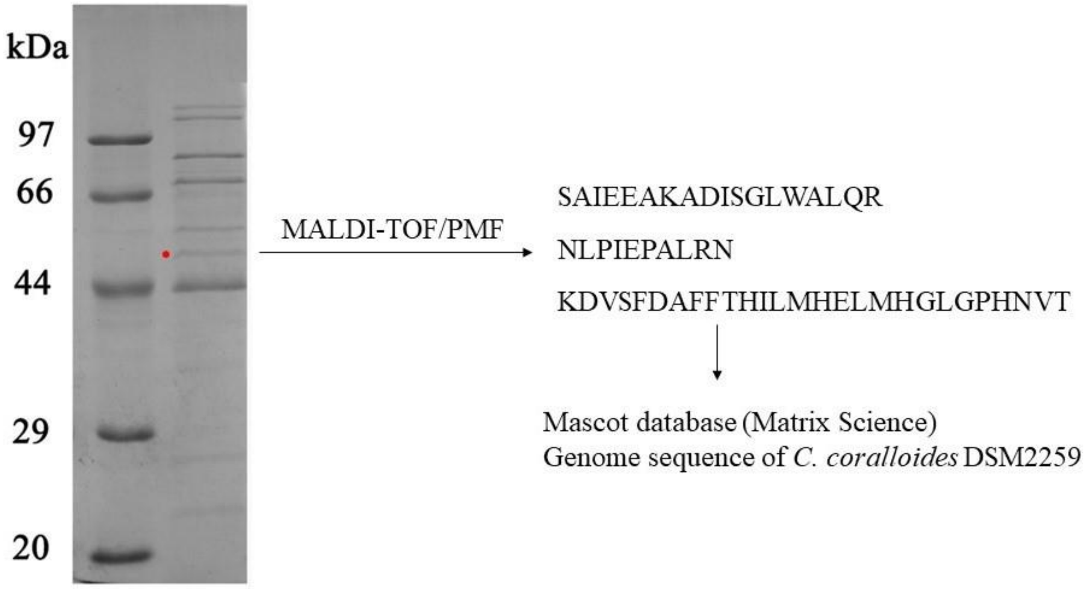

Figure. S1 The MALDI-TOF result of the extracellular protein from *Corallococcus* sp. EGB

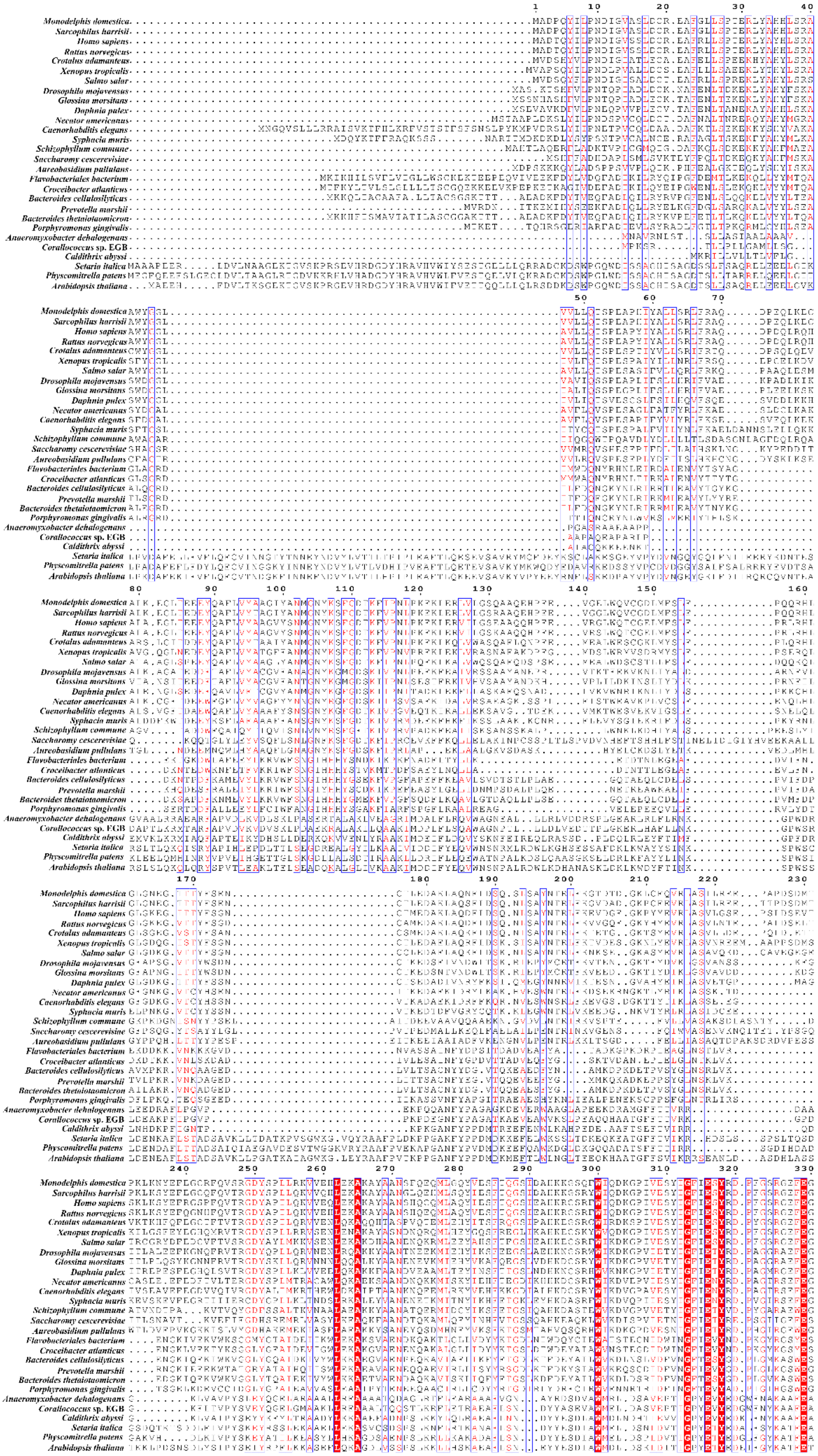
Monodelphis domestica
Sarcophilus harrisii
Homo sapiens
Rattus norvegicus
Crotalus adamanteus
Xenopus tropicalis
Salmo salar
Drosophila mojavensus
Glossina morsitans
Daphnia pulex
Necator americanus
Caenorhabditis elegans
Syphacia muris
Schizophyllum commune
Saccharomy cescerevisiae
Aureobasidium pullulans
Flavobacteriales bacterium
Croceibacter atlanticus
Bacteroides cellulosilyticus
Prevotella marshii
Bacteroides thetaiotaomicron
Porphyromonas gingivalis
Anaeromyxobacter dehalogenans
Corallococcus sp. EGB
Caldithrix abyssi
Setaria italica
Physcomitrella patens
Arabidopsis thaliana

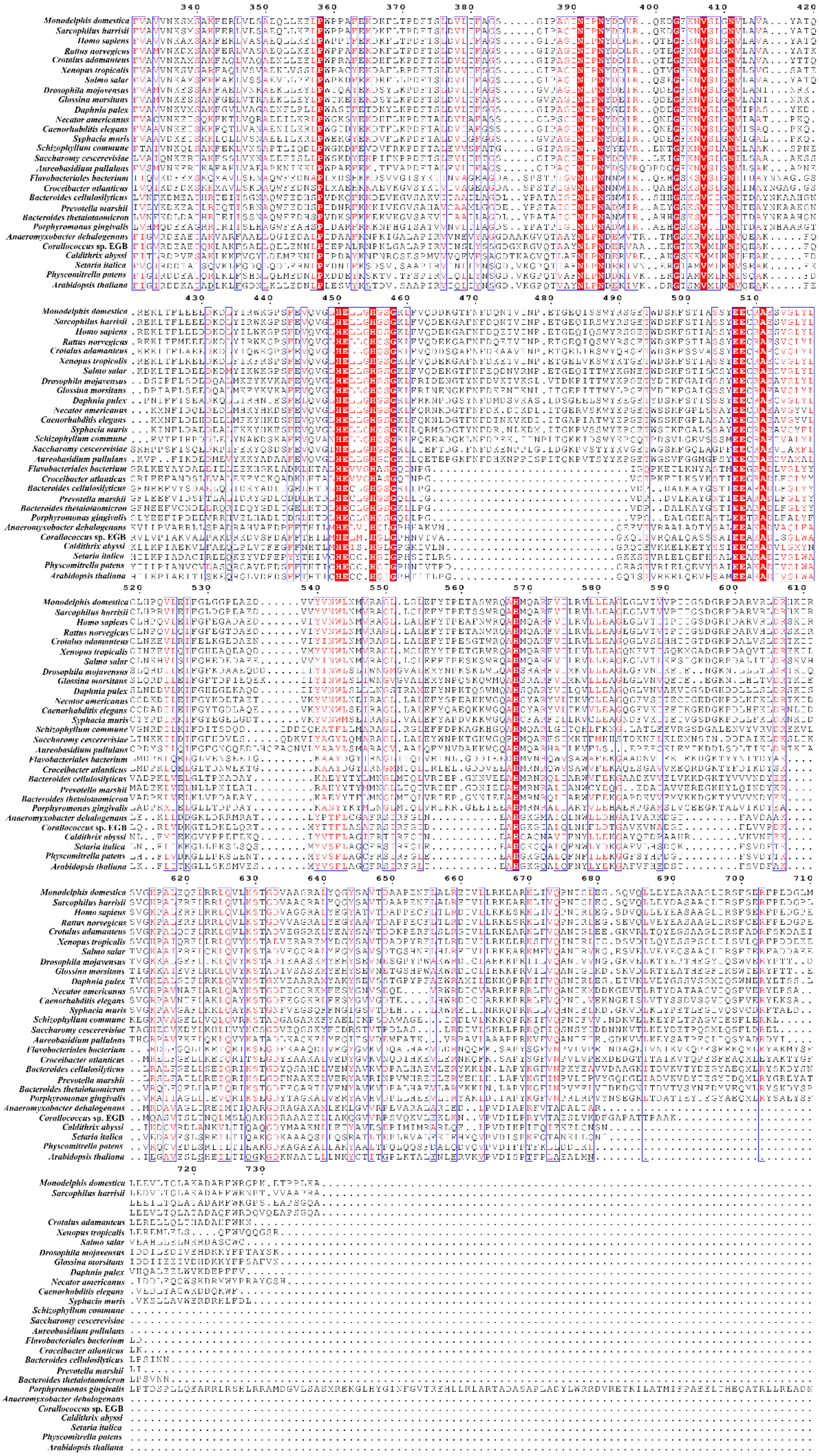
Monodelphis domestica
Sarcophilus harrisii
Homo sapiens
Rattus norvegicus
Crotalus adamanteus
Xenopus tropicalis
Salmo salar
Drosophila mojavensus
Glossina morsitans
Daphnia pulex
Necator americanus
Caenorhabditis elegans
Syphacia muris
Schizophyllum commune
Saccharomy cescerevisiae
Aureobasidium pullulans
Flavobacteriales bacterium
Croceibacter atlanticus
Bacteroides cellulosilyticus
Prevotella marshii
Bacteroides thetaiotaomicron
Porphyromonas gingivalis
Anaeromyxobacter dehalogenans
Corallococcus sp. EGB
Caldithrix abyssi
Setaria italica
Physcomitrella patens
Arabidopsis thaliana

Figure. S2 Multiple sequence alignment of DPP III from various species. The organisms and UniProt sequence identifiers are *Monodelphis domestica* (F6ZXH8), *Sarcophilus harrisii* (A0A7N4P2W4), *Homo sapiens* (Q9NY33), *Rattus norvegicus* (O55096), *Crotalus adamanteus* (J3RZ30), *Xenopus laevis* (Q6DE90), *Salmo salar* (B5X435), *Drosophila mojavensis* (B4K999), *Glossina morsitans* (D3TMQ7), *Daphnia pulex* (E9GTX0), *Necator americanus* (W2SLT7), *Caenorhabditis elegans* (G5ECW7), *Syphacia muris* (A0A0N5AXJ5), *Schizophyllum commune* (D8PVF6), *Saccharomyces cerevisiae* (Q08225), *Aureobasidium pullulans* (A0A074XX23), *Flavobacteriales bacterium* (A4AQA4), *Croceibacter atlanticus* (A3U8C5), *Bacteroides cellulosilyticus* (E2NMV7), *Prevotella marshii* (E0NQE0), *Bacteroides thetaiotaomicron* (A0A139KC05), *Porphyromonas gingivalis* (Q7MX92), *Anaeromyxobacter dehalogenans* (Q2IN78), *Corallococcus* sp. EGB (A0A5H1ZR28), *Caldithrix abyssi* (H1XW48), *Setaria italica* (K3YPW2), *Physcomitrella patens* (A9TLP4), *Arabidopsis thaliana* (Q8L831). Dots indicate gaps. Identical amino acid residues at the same position are shaded.

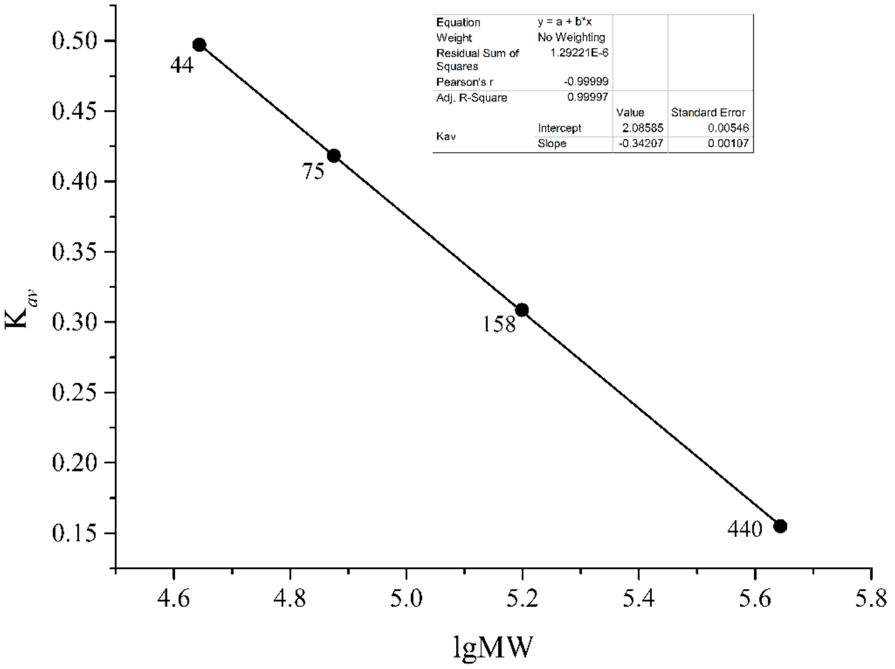

Figure. S3 The calibration curve lgMW *vs* K*av*. The equation is K*av* =2.08585-0.34207lgMW, where the K*av* is distribution coefficient, MW is the molecular weight of the standard samples. The V_e, K*av* and lgMW for *Co*DPP III is 71.95 mL, 0.3513 and 5.0708, respectively.

Table S1 Effect of metal ions on *Co*DPP III activity

Metal ions	Relative activity (%)		
	0.1 mM	0.5 mM	1.0 mM
No addition	100	100	100
Zn^{2+}	78.15±0.6	10.6±0.1	-
Co^{2+}	141.06±1.2	121.19±2.2	105.2±2.4
Mn^{2+}	129.14±3.3	111.92±2.1	98.92±2.1
Mg^{2+}	119.21±1.6	129.8±2.7	109.7±1.5
Ba^{2+}	121.19±0.9	123.18±1.3	105.93±1.2
Ca^{2+}	116.56±0.8	121.19±2.1	114.21±2.1
Cu^{2+}	4.64±0.1	-	-
Ni^{2+}	7.95±0.2	5.96±0.1	-

Table S2 Strains and plasmids used in this study

Strains and plasmids	Relevant genotype or characteristic(s)	Reference
Corallococcus sp. EGB	wild type	[45]
E. coli		
E. coli BL21(DE3)	F^- *ompT hsdS* (r_B^- m_B^-) *gal dcm lacY1*(DE3)	Novagen
E. coli B834(DE3)	F^- *ompT hsdS* (r_B^- m_B^-) *gal dcm met* (DE3)	Novagen
M. xanthus		
DK1622	wild type	[46]
DK1622-*Codpp* III	*attB*::pET29a-*attp-Codpp* III	This study
DK1622-△*asgA*	DK1622 mutant with *asgA* gene disrupted	This study
**Plasmids**		
pET29a	pBR322 ori, Kan^r	Novagen
pBJ113	pBR322 ori, galK; Kan^r	[47]
pET29a-*Codpp* III	*Codpp* III inserted into *Nde* I/*Xho* I site of pET29a	This study
pET29a-*attP*	pET29a derivative carrying the MX8 *attP* region, Kan^r	This study
pET29a-*attp-Codpp* III	A 2.7 kb fragment containing *Codpp III gene and its promoter inserted into Hind* III/*Xho* I site of pET29a-*attP*	This study
pBJ113-*asgA*	1073 bp internal fragment of *asgA* inserted into *Hind* III/*EcoR* I site of pBJ113	This study
